# Long-term outcomes of vedolizumab in inflammatory bowel disease: the Swedish prospective multicentre SVEAH extension study

**DOI:** 10.1177/17562848231174953

**Published:** 2023-05-30

**Authors:** Isabella Visuri, Carl Eriksson, Sara Karlqvist, Byron Lykiardopoulos, Per Karlén, Olof Grip, Charlotte Söderman, Sven Almer, Erik Hertervig, Jan Marsal, Carolina Malmgren, Jenny Delin, Hans Strid, Mats Sjöberg, Daniel Bergemalm, Henrik Hjortswang, Jonas Halfvarson

**Affiliations:** Department of Gastroenterology, Faculty of Medicine and Health, Örebro University, Södra Grev Rosengatan 30, Örebro, SE-70182 Sweden; Department of Gastroenterology, Faculty of Medicine and Health, Örebro University, Örebro, Sweden; Clinical Epidemiology Division, Department of Medicine Solna, Karolinska Institutet, Stockholm, Sweden; Department of Gastroenterology, Faculty of Medicine and Health, Örebro University, Örebro, Sweden; Department of Gastroenterology and Hepatology, Linköping University, Linköping, Sweden; Department of Internal Medicine, Danderyd Hospital, Stockholm, Sweden; Department of Gastroenterology, Skåne University Hospital, Malmö/Lund, Sweden; Department of Internal Medicine, St Göran Hospital, Stockholm, Sweden; Department of Medicine Solna, Division of Gastroenterolgy, Karolinska Institutet, Stockholm, Sweden; IBD-Unit, Division of Gastroenterology, Karolinska University Hospital, Stockholm, Sweden; Department of Gastroenterology, Skåne University Hospital, Malmö/Lund, Sweden; Department of Gastroenterology, Skåne University Hospital, Malmö/Lund, Sweden; Takeda Pharma AB, Stockholm, Sweden; Department of Gastroenterology, Ersta Hospital, Stockholm, Sweden; Department of Internal Medicine, Södra Älvsborgs Hospital, Borås, Sweden; Department of Internal Medicine, Skaraborgs Hospital, Lidköping, Sweden; Department of Gastroenterology, Faculty of Medicine and Health, Örebro University, Örebro, Sweden; Department of Gastroenterology and Hepatology, Linköping University, Linköping, Sweden; Department of Health, Medicine, and Caring Sciences, Linköping University, Linköping, Sweden; Department of Gastroenterology, Faculty of Medicine and Health, Örebro University, Örebro, Sweden

**Keywords:** Crohn’s disease, inflammatory bowel disease, real-world data, ulcerative colitis, vedolizumab

## Abstract

**Background:**

Real-world data on long-term outcomes of vedolizumab (VDZ) are scarce.

**Objective:**

To assess long-term outcomes (up to 3 years) of VDZ in treating inflammatory bowel disease (IBD).

**Design:**

A nationwide, prospective multicentre extension of a Swedish observational study on VDZ assessing Effectiveness And Healthcare resource utilization in patients with IBD (SVEAH).

**Methods:**

After re-consent, data of patients with Crohn’s disease (CD) (*n* = 68) and ulcerative colitis (UC) (*n* = 46) treated with VDZ were prospectively recorded using an electronic case report form integrated with the Swedish IBD Register (SWIBREG). The primary outcome was clinical remission (defined as Harvey–Bradshaw Index ⩽4 in CD and partial Mayo score ⩽2 in UC) at 104 and 156 weeks in patients with a response and/or remission 12 weeks after starting VDZ. Secondary outcomes included health-related quality of life (HRQoL) and biochemical outcomes.

**Results:**

VDZ continuation rates were high at weeks 104 and 156, 88% and 84%, respectively, for CD and 87% and 78%, respectively, for UC. Of the 53 CD patients with a response/remission at 12 weeks, 40 (75%) patients were in remission at 104 weeks and 42 (79%) patients at 156 weeks. For UC, these numbers were 25/31 (81%) and 22/31 (71%), respectively. Improvements were seen in the Short Health Scale (*p* < 0.01 for each dimension; CD, *n* = 51; UC, *n* = 33) and the EuroQol 5-Dimensions, 5-levels index value (*p* < 0.01; CD, *n* = 39; UC, *n* = 30). Median plasma-C-reactive protein concentrations (mg/L) decreased from 5 at baseline to 4 in CD (*p* = 0.01, *n* = 53) and from 5 to 4 in UC (*p* = 0.03, *n* = 34) at 156 weeks. Correspondingly, median faecal-calprotectin (µg/g) decreased from 641 to 114 in CD patients (*p* < 0.01, *n* = 26) and from 387 to 37 in UC patients (*p* = 0.02, *n* = 17).

**Conclusion:**

VDZ demonstrated high continuation rates and was associated with improvements in clinical outcomes, HRQoL measures and inflammatory markers at 2 and 3 years after treatment initiation in this prospective national SVEAH extension study.

**Registration:**

ENCePP registration number: EUPAS22735.

## Introduction

Inflammatory bowel disease (IBD) is a progressive chronic inflammatory disease subdivided into two main subtypes, Crohn’s disease (CD) and ulcerative colitis (UC). Treatment aims to induce and maintain remission to prevent disease progression, including hospitalizations, surgery and worsening of health-related quality of life (HRQoL).^1,2^ In patients with failure or intolerance to conventional treatment, anti-tumour necrosis factor (anti-TNF) agents have become the mainstay of treatment. However, more than one-third of the patients treated with anti-TNF agents do not respond initially or lose response to therapy.^3–8^ Consequently, therapies with alternative mechanisms of action are needed.

Vedolizumab (VDZ) is a monoclonal antibody directed towards the integrin α4β7. T-cells expressing α4β7 migrate predominantly to the gastrointestinal tract and gut-associated lymphoid tissue through adhesion to mucosal addressin cell adhesion molecule-1 (MAdCAM-1). By blocking the interaction between α4β7 and MAdCAM-1, VDZ seems to induce gut selective immunosuppression, although additional local and systemic effects have been proposed.^9,10^ VDZ was approved for the treatment of moderately to severely active CD and UC by the European Medical Agency and the U.S. Food and Drug Administration in 2014 based on the GEMINI studies.^11–13^ In these phase III randomized controlled trials (RCTs), patients with CD or UC were treated with VDZ for up to 1 year.^11–13^

However, because IBD is a chronic disease, patients usually require long-term maintenance treatment. Loftus *et al.* reported a favourable safety and efficacy profile of VDZ in the GEMINI long-term safety (LTS) study, with median cumulative exposure periods of 42.4 months in patients with UC and 31.5 months in CD. Most of the patients in the GEMINI LTS study were recruited from GEMINI 1 and 2.^14^ Whether these long-term findings can be generalized to IBD patients treated in routine practice is an open question, mainly because many patients are not eligible for inclusion in RCTs.^15,16^ Real-world outcomes of VDZ treatment have been reported in several observational studies.^17,18^ However, most previously conducted studies have reported outcomes after a short period,^19,20^ lack data on HRQoL outcomes,^21^ or are based on a retrospective design.^22,23^ Other limitations include the inclusion of patients at tertiary referral centres^24^ or from regional cohorts.^25,26^ In our previous nationwide prospective Swedish observational study on VDZ assessing Effectiveness And Healthcare resource utilization in patients with IBD (SVEAH), we followed patients treated with VDZ for 1 year and reported clinical outcomes, including HRQoL.^27^ To our knowledge, only two real-world studies have followed patients prospectively and reported remission rates beyond 1 year.^28,29^

To examine the long-term clinical effectiveness and safety of VDZ, we extended the nationwide SVEAH study and assessed outcomes at 2 and 3 years of treatment by analysing prospectively and systematically collected real-world data in the Swedish IBD Register (SWIBREG).

## Materials and methods

### Study design

This study was a nationwide prospective multicentre observational extension study of patients with CD and UC, designed to assess the long-term effectiveness and safety of VDZ in clinical practice. Patients from the SVEAH study,^27^ who were still treated with VDZ at week 52, were eligible for inclusion. After re-consent, patients were prospectively followed, with annual visits scheduled at weeks 104 and 156 (±8 weeks). The reporting of this study conforms to the Strengthening the Reporting of Observational Studies in Epidemiology statement.^30^

### Patient population

The SVEAH study population has been described elsewhere.^27^ In short, patients aged ⩾18 years with active disease at initiation of treatment with VDZ, were recruited from 21 hospitals between 2015 and 2018. Active CD was defined as the presence of symptoms, based on patient-reported outcomes, accompanied by ulcers at colonoscopy or signs of active inflammation at magnetic resonance imaging, increased plasma-C-reactive protein (P-CRP) or P-high sensitivity-CRP >2.87 mg/L or faecal-calprotectin (f-calprotectin) >200 µg/g. Normal P-CRP was considered as a value below the lower limit of detection, generally <3.0–5.0 mg/L. Active UC was defined as the combination of symptoms and a Mayo Clinic Endoscopic Subscore ⩾2. Patients with previous exposure to VDZ or contraindications to the drug were excluded.

Of the 21 hospitals participating in the SVEAH study, 13 (8 regional and 5 university hospitals) took part in the SVEAH extension study. Patients still treated with VDZ at week 52 were eligible for inclusion.

Maintenance treatment with VDZ was administered intravenously. No predetermined dosing schedule was applied; doses and dosing intervals were at the discretion of the treating physicians.

### Data collection

An electronic case report form (eCRF), integrated with the SWIBREG, was used to collect study data systematically. Patients were identified using the identification number assigned when registered in SWIBREG. The data collection process has previously been described.^27^ Information about basic demographics, clinical characteristics according to the Montreal classification,^31^ disease activity, HRQoL, previous anti-TNF treatment and concomitant use of other IBD treatments at baseline (defined as the start of VDZ treatment) was recorded. Data on treatments, potential adverse drug reaction (ADR), clinical activity measured by the Harvey–Bradshaw Index (HBI) for CD and the Mayo Clinic score for UC, respectively,^32,33^ biochemical measures (B-haemoglobin (B-Hb), P-CRP and f-calprotectin) and endoscopic activity were recorded. Concomitant corticosteroid treatment was defined as oral or intravenous betamethasone, oral prednisolone or budesonide treatment. Reasons for termination of the last anti-TNF treatment were primary non-response, loss of response (defined as recurrence of symptoms during scheduled maintenance dosing), intolerance and other reasons. Reasons for termination of VDZ therapy were evaluated based on categories used in SWIBREG, that is, lack or loss of response, ADR and other reasons (pregnancy and patient’s request to withdraw).

HRQoL was assessed with a general life quality index, the EuroQol 5-Dimensions, 5-levels (EQ-5D-5L) index and the IBD-specific Short Health Scale (SHS). The EQ-5D-5L index consists of five dimensions (mobility, self-care, usual activities, pain/discomfort and anxiety/depression)^34^ and the SHS comprises four dimensions (symptoms, function, anxiety and well-being).^35^ Safety reporting was performed by the investigators as required by the national law for approved medicinal products, including any ADR or serious adverse event (SAE). The form used by the Swedish Medical Product Agency was implemented in the eCRF to facilitate this process, and the local investigator sent reports to the Swedish Medical Products Agency and to Takeda, Sweden.

### Outcomes

The primary outcome was clinical remission at 104 and 156 weeks in patients with response or remission 12 weeks after initiation of VDZ treatment. Clinical remission was defined as a HBI ⩽4 in CD and as a partial Mayo Clinic score (pMayo Clinic score) ⩽2 in UC. Clinical response was expressed in CD as a decrease of ⩾3 points from baseline in the HBI and a drop in the pMayo Clinic score of ⩾2 points with a reduction of at least 25% from baseline in UC, with a decrease of ⩾1 point on the rectal bleeding score or an absolute rectal bleeding score of 0 or 1. Baseline was defined as the initiation of VDZ therapy.

Secondary outcomes were clinical response at weeks 104 and 156; sustained remission at weeks 52, 104 and 156; in week 12 responders, clinical response and remission in all included patients, corticosteroid-free clinical remission and drug continuation rates at weeks 104 and 156.

Other secondary outcomes included endoscopic remission, HRQoL, change in the presence of extraintestinal symptoms, B-Hb, P-CRP, f-calprotectin and surgical resections. Safety outcomes encompassed ADRs and SAEs captured during the study period and spontaneously reported. ADRs and SAEs of special interest were predefined as malignancy and infections requiring antibiotics.

### Statistics

Continuous variables are described as the number of observations and median (interquartile range, (IQR)). Categorical variables are expressed as frequencies and percentages. Differences between groups were assessed with the non-parametric Mann-Whitney U test or chi-square test where appropriate.

We applied an intention-to-treat approach and reported remission and response rates were based on non-responder imputation where missing data and discontinuation of VDZ were considered as treatment failure, regardless of the reason for discontinuation. For comparisons of f-calprotectin, P-CRP, B-Hb, HBI, pMayo Clinic score, SHS and EQ-5D-5L index values between baseline and follow-up, we performed paired assessments using the Wilcoxon matched-pairs signed-rank test. For clarity, the number of individuals with valid data is reported in brackets for each analysis. When up to two items were missing for the SHS and EQ-5D-5L index value (*n* = 1), these measures were imputed to the mean value. The EQ-5D-5L index calculator, provided by the EuroQol group, was used to calculate the EQ-5D-5L index values. Survival plots were generated using the Kaplan–Meier analyses. In the survival analyses, baseline was set at week 52. In the analyses of corticosteroid-free remission rates, only patients who received concomitant treatment with corticosteroids at the initiation of VDZ therapy were included. Extraintestinal manifestations were treated as a categorical outcome (present or absent). Multivariable logistic regression models were constructed to identify potential predictors of clinical remission at week 156 in patients with CD and UC. Patients who discontinued the VDZ therapy before week 52 and the patients included in this extension study were included. To avoid potential bias because of missing data, patients who continued VDZ treatment beyond week 52 but did not participate in the extension study were excluded. The included variables were selected on their potential biological association with the outcome. Variables with <10 cases for each category were excluded in the statistical analyses. Disease duration, HBI and pMayo Clinic score at baseline were treated as continuous measures, whereas sex, disease location, behaviour, disease extent, extraintestinal manifestations, previous surgery, concurrent medication and reasons for termination of last anti-TNF were used as categorical measures.

To examine potential inclusion bias, we compared patients participating in the SVEAH extension study with those who were eligible but did not participate in the extension.

All variables were subject to systematic verification using queries to ensure correct observations. A *p*-value of <0.05 or a 95% confidence interval (95% CI) not including 1.00 was considered statistically significant. Because there was no comparison group in this study, a formal power calculation was not performed. As previously described, the sample size was based on the feasibility of providing reasonable precision in outcome estimates.^27^ Statistical analyses were executed using SPSS Statistics for Windows version 27.0 (IBM Corp., Armonk, NY, USA, 2017).

## Results

### Patient population

**Figure 1.**
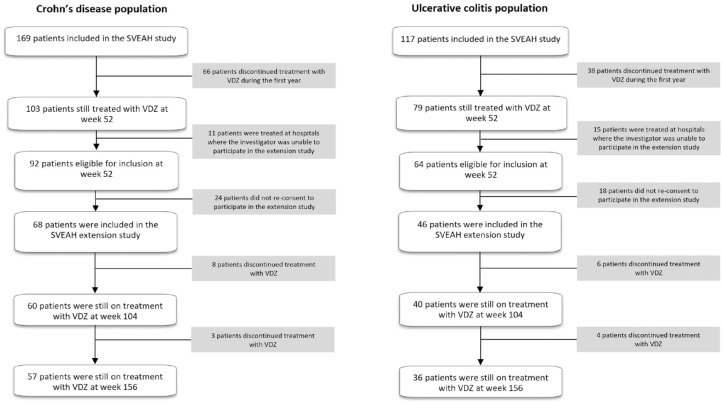
Flowchart of the SVEAH extension study. SVEAH, a Swedish observational study on vedolizumab assessing Effectiveness And Healthcare resource utilization in patients with inflammatory bowel disease.

In total, 103/169 patients (61%) with CD and 79/117 patients (68%) with UC in the original SVEAH study remained on VDZ treatment after 52 weeks. Of these patients, 92 patients with CD and 64 with UC were eligible for inclusion in the SVEAH extension (Figure 1). After obtaining a written informed consent, 68 (74%) patients with CD and 46 (72%) patients with UC were included. Demographics and clinical characteristics at baseline are presented in Table 1.

**Table 1.** Demographics and clinical characteristics at the initiation of VDZ treatment in patients with CD and UC included in the SVEAH extension study.

	CD (*n* = 68)	UC (*n* = 46)
Female sex, *n* (%)	27 (40)	23 (50)
Median age, years (IQR)	43 (31–53)	42 (26–53)
Disease duration, years (IQR)	9 (3–23)	6 (3–11)
Smoker, *n* (%)	8 (12)	2 (4)
Disease location, *n* (%)		
Ileal, L1	16 (24)	
Colonic, L2	24 (35)	
Ileocolonic, L3	28 (41)	
Isolated upper disease, L4	0 (0)	
Disease behaviour, *n* (%)		
Inflammatory, B1	42 (62)	
Stricturing, B2	21 (31)	
Penetrating, B3	5 (7)	
Perianal, p	13 (19)	
Disease extent, *n* (%)		
Proctitis, E1		1 (2)
Left-sided colitis, E2		9 (20)
Extensive colitis, E3		36 (78)
Previous biologics, *n* (%)		
0	10 (15)	4 (9)
1	24 (35)	28 (61)
⩾2	34 (50)	14 (30)
Reason for termination of last biological treatment, *n* (%)		
Primary non-response	13 (22)	15 (36)
Loss of response	27 (47)	19 (45)
Intolerance	15 (26)	8 (19)
Other reasons	3 (5)	
Previous IBD surgery, *n* (%)	27 (40)	2 (4)^a^
Concomitant medication, *n* (%)		
5-aminosalicylic acid	4 (6)	20 (43)
Corticosteroids	14 (21)	11 (24)
Immunomodulators	10 (15)	11 (24)

a Colectomy with ileorectal anastomosis.

IBD, inflammatory bowel disease; IQR, interquartile range; SVEAH, a Swedish observational study on VDZ assessing Effectiveness And Healthcare resource utilization in patients with inflammatory bowel disease; VDZ, vedolizumab.

### Crohn’s disease

**Figure 2.**
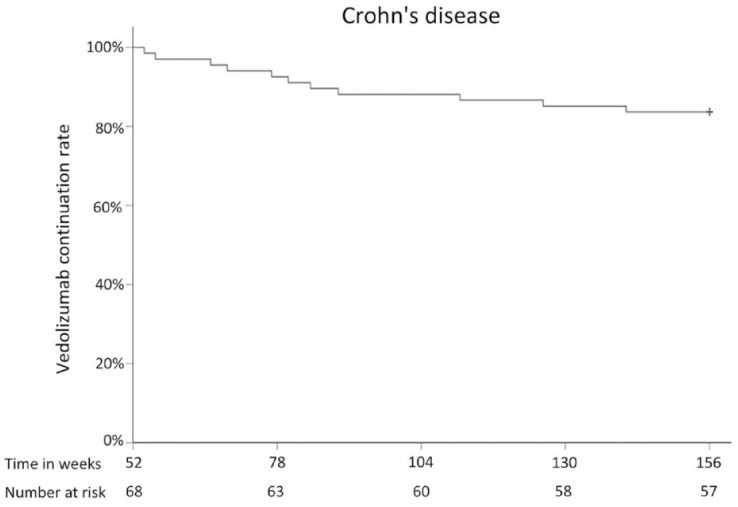
VDZ continuation rate in patients with CD. CD, Crohn’s disease; VDZ, vedolizumab.

Some 60 CD patients were still treated with VDZ at 104 weeks after treatment initiation and 57 CD patients at 156 weeks, corresponding to a continuation rate of 88% (95% CI: 81–96%) at week 104 and 84% (95% CI: 75–93%) at week 156 (Figure 2). Reasons for discontinuation were loss of response (*n* = 10) and ADRs (*n* = 1). Two patients underwent CD-associated surgery: intestinal fistula surgery after 64 weeks (*n* = 1) and perianal fistula surgery after 131 weeks (*n* = 1). During the SVEAH extension, that is, between weeks 52 and 156, 46 (68%) patients continued maintenance treatment with 300 mg VDZ intravenously every 8 weeks, 11 (16%) shortened the infusion interval, 5 (7%) extended the interval and 6 (9%) were treated with various combinations of shorter and longer infusion intervals.

### Clinical outcomes

**Figure 3.**
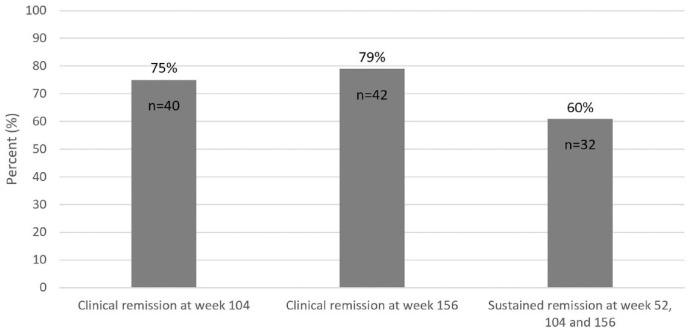
Clinical remission rates at weeks 104 and 156, among CD patients (*n* = 53) with a clinical response or remission at 12 weeks after the initiation of VDZ treatment. Missing data and discontinuation of VDZ were assumed to represent treatment failure. CD, Crohn’s disease; VDZ, vedolizumab.

For CD patients with a response or remission to VDZ at week 12 (*n* = 53), 40 (75%) patients were in clinical remission (defined as an HBI ⩽4) at week 104; 42 (79%) patients at weeks 156 and 32 (60%) patients were in sustained clinical remission at weeks 52, 104 and 156 (Figure 3). An additional 6 (11%) and 2 (4%) patients were still on VDZ treatment at weeks 104 and 156, respectively, but lacked information on HBI within the pre-specified time-window.

**Figure 4.**
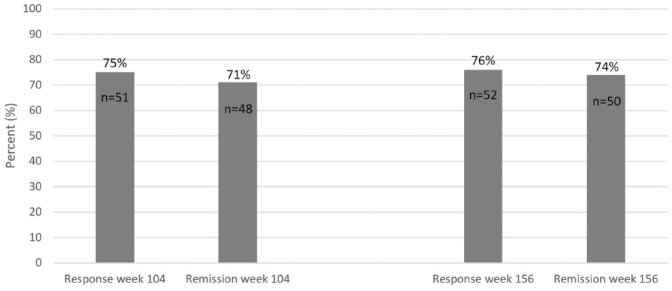
Clinical response and remission rates at weeks 104 and 156 in all CD patients (*n* = 68) in the SVEAH extension study. Missing data and discontinuation of VDZ were assumed to represent treatment failure. CD, Crohn’s disease; SVEAH, a Swedish observational study on vedolizumab assessing Effectiveness And Healthcare resource utilization in patients with inflammatory bowel disease.

Of all CD patients (*n* = 68) in the SVEAH extension study, 51 (75%) patients had a clinical response and 48 (71%) patients were in clinical remission at week 104 (Figure 4). Correspondingly, 52 (76%) patients had a clinical response and 50 (74%) patients were in clinical remission at week 156. Among patients still treated with VDZ at these follow-ups, information on clinical response and remission status was missing within the pre-specified time-window for six patients (9%) at week 104 and three patients (4%) at week 156. For patients with concomitant systemic corticosteroids at baseline (*n* = 14), 9 (64%) patients were in corticosteroid-free remission at week 104 and 5 (36%) patients at week 156. All 68 CD patients in the SVEAH extension study had an HBI score at baseline, and 54/60 (90%) patients also had valid HBI scores at week 104 and 54/57 (95%) patients at week 156. Compared to baseline, the median (IQR) HBI score decreased from 5 (2–9) to 2 (0–3) at week 104 (*p* < 0.001) and from 5 (2–8) to 1 (0–2) at week 156 (*p* < 0.001).

### Biochemical outcomes

Information about P-CRP was available for all patients at baseline, 54 (90%) patients also had valid P-CRP values at week 104 and 53 (93%) at 156. Another 6 (10%) patients still on VDZ lacked P-CRP data within the pre-specified time-window at week 104 and 4 (7%) patients at week 156. Compared to baseline, the median (IQR) P-CRP levels (mg/L) decreased from 5 (2–14) to 4 (1–9) at week 104 (*p* = 0.08, *n* = 54) and from 5 (2–16) to 4 (2–7) at week 156 (*p* = 0.01, *n* = 53). Correspondingly, the median (IQR) f-calprotectin concentrations (µg/g) decreased from 641 (254–1190) to 140 (66–560) at week 104 (*p* = 0.01, *n* = 22) and from 641 (242–1189) to 114 (44–291) at week 156 (*p* < 0.001, *n* = 26). The median (IQR) B-Hb concentration (g/L) did not statistically significantly change between baseline (135 (124–146)) and week 104 (137 (128–145); *p* = 0.38, *n* = 55), but increased significantly from 135 (126–147) at baseline to 141 (134–152) at week 156 (*p* = 0.02, *n* = 53).

### Endoscopic outcomes

Among CD patients (*n* = 68), 15 (22%) were in endoscopic remission, defined as the absence of ulcers, at week 104 and 13 (19%) at week 156. However, data on endoscopic activity were missing in 45 patients (66%) at week 104 and 43 (63%) at week 156. Therefore, we repeated the analyses and included patients with information on endoscopic outcomes at the follow-up visits only. Among these patients, 100% (15/15) were in endoscopic remission at week 104 and 93% (13/14) at week 156.

### HRQoL outcomes

All 68 CD patients had information about SHS and EQ-5D-5L at baseline: 54 patients also had valid data on SHS at week 104 and 51 at week 156. Compared to baseline, the SHS improved significantly in all dimensions at weeks 104 and 156 (Table 2). Correspondingly, the median (IQR) EQ-5D-5L index value increased from 0.77 (0.73–0.86) at baseline to 0.86 (0.77–1.0) at week 104 (*p* = 0.001, *n* = 39) and to 0.86 (0.81–1.0) at week 156 (*p* < 0.001, *n* = 39).

**Table 2.** HRQoL at baseline, at the start of VDZ treatment, at 104 and 156 weeks in patients with CD.

	Baseline	Week 104	*p*-value	Week 156	*p*-value
	Median (IQR)	Median (IQR)		Median (IQR)	
SHS					
Bowel symptoms	2 (1–3)	1 (0–2)	<0.001	1 (0–1)	0.002
Activities of daily living	2 (1–3)	1 (0–1)	<0.001	1 (0–1)	<0.001
Worry	2 (1–3)	1 (1–2)	<0.001	1 (0–1)	<0.001
General well-being	2 (1–3)	1 (1–2)	<0.001	1 (1–2)	<0.001
EQ-5D-5L					
Mobility	1 (1–1)	1 (1–1)	0.08	1 (1–1)	0.02
Self-care	1 (1–1)	1 (1–1)	0.32	1 (1–1)	0.32
Usual activities	1 (1–2)	1 (1–1)	0.02	1 (1–1)	0.002
Pain/discomfort	2 (2–3)	1 (1–2)	0.001	1 (1–2)	0.001
Anxiety/depression	2 (1–2)	2 (1–2)	0.04	1 (1–2)	<0.001
Visual analogue scale	70 (50–75)	80 (75–90)	<0.001	85 (79–90)	<0.001
EQ-5D-5L index value	0.77 (0.73–0.86)	0.86 (0.77–1.0)	0.001	0.86 (0.81–1.0)	<0.001

Comparisons between baseline and at weeks 104 (*n* = 39) and 156 (*n* = 39) were performed using the Wilcoxon matched-pairs signed-rank test.

CD, Crohn’s disease; EQ-5D-5L, EuroQol 5-Dimensions 5-levels; HRQoL, health-related quality of life; IQR, interquartile range; SHS, Short Health Scale; VDZ, vedolizumab.

### Extraintestinal manifestations

Extraintestinal manifestations were observed in 11 CD patients (arthritis/arthralgia, *n* = 11) at baseline, 5 (arthralgia *n* = 4; primary sclerosing cholangitis *n* = 1) at week 104 and 7 (arthritis/arthralgia *n* = 6; primary sclerosing cholangitis *n* = 1) at week 156.

### Predictors of remission

Univariate and multivariable logistic regression analyses were performed to identify predictors of clinical remission at week 156 (Table 3). Patients who discontinued vedolizumab before week 52 and the patients included in this extension study were included and are presented in Supplementary Table 1. After adjustment for the potential confounders listed in Table 3, a high HBI (OR: 0.87; 95% CI: 0.78–0.96) was the only clinical variable at initiation of vedolizumab associated with clinical remission at week 156.

**Table 3.** Predictors of clinical remission at week 156 in patients with CD.

	Univariate analysis	Multivariable analysis
Female sex	**0.46 (0.23–0.94)**	0.65 (0.28–1.50)
Disease duration at baseline	0.99 (0.96–1.02)	1.01 (0.97–1.06)
HBI at baseline	**0.85 (0.78–0.93)**	**0.87 (0.78–0.96)**
Location		
Ileal (L1)	Ref	Ref
Colonic (L2)	1.23 (0.59–2.54)	0.99 (0.25–3.94)
Ileocolonic (L3)	0.75 (0.37–1.51)	1.14 (0.33–3.93)
Behaviour		
Inflammatory (B1)	Ref	Ref
Stricturing (B2)	0.70 (0.33–1.50)	0.84 (0.25–2.81)
Penetrating (B3)	0.83 (0.24–2.90)	0.24 (0.03–1.92)
Perianal disease (p)	0.99 (0.41–2.36)	3.22 (0.75–13.78)
Extraintestinal manifestations at baseline	0.41 (0.16–1.03)	0.55 (0.18–1.71)
Previous surgery	0.49 (0.24–1.03)	0.47 (0.14–1.58)
Concurrent medication at baseline		
Corticosteroids	1.06 (0.44–2.57)	0.99 (0.33–2.99)
Immunomodulators	2.42 (0.84–6.96)	2.71 (0.74–10.0)
Reasons for termination of last anti-TNF		
Anti-TNF naive	Ref	Ref
Primary non-response	1.15 (0.47–2.80)	0.53 (0.12–2.38)
Secondary loss of response/ADR/other	0.58 (0.27–1.23)	0.29 (0.08–1.05)

Statistically significant results are highlighted in bold. ADR, adverse drug reaction; Anti-TNF, anti-tumour necrosis factor; CD, Crohn’s disease; HBI, Harvey–Bradshaw Index.

### Safety outcomes

Two events were reported in a 42-year-old male patient who developed pulmonary embolism after 2 months of VDZ treatment and was diagnosed with bladder cancer during the subsequent year.

### Ulcerative colitis

**Figure 5.**
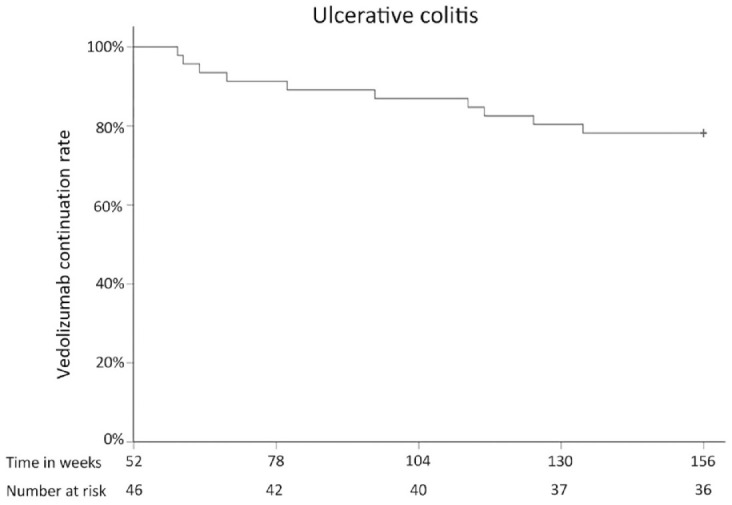
VDZ continuation rate in patients with UC. UC, ulcerative colitis; VDZ, vedolizumab.

Some 40 UC patients who had been included in the SVEAH extension study were still treated with VDZ at 104 weeks after treatment initiation and 36 at 156 weeks (Figure 5), corresponding to a VDZ continuation rate of 87% (95% CI: 77%–97%) at week 104 and 78% (95% CI: 66%–90%) at week 156. Reasons for discontinuation were lack/loss of response (*n* = 7), ADR (*n* = 1), pregnancy (*n* = 1) and patient request (*n* = 1). One patient underwent surgery (drainage of abscess) after 137 weeks of treatment with VDZ. During the SVEAH extension, that is, between weeks 52 and 156, 36 (78%) patients with UC continued maintenance treatment with 300 mg VDZ intravenously every 8 weeks, 2 (4%) UC patients shortened the infusion interval, 3 (7%) extended the interval and 5 (11%) were treated with various combinations of shorter and longer infusion intervals.

### Clinical outcomes

**Figure 6.**
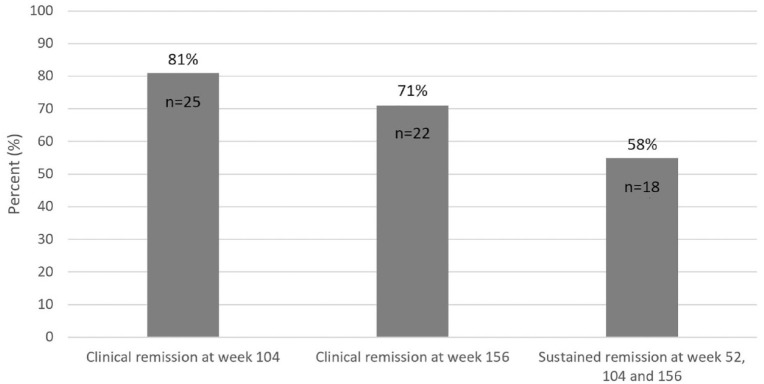
Clinical remission rates at weeks 104 and 156 among UC patients (*n* = 31) with a clinical response or remission at 12 weeks after initiation of VDZ treatment. Missing data and discontinuation of VDZ were assumed to represent treatment failure. UC, ulcerative colitis; VDZ, vedolizumab.

For UC patients with a response or remission to VDZ at week 12 (*n* = 31), 25 (81%) were in clinical remission (defined as a pMayo Clinic score ⩽2) at week 104, 22 (71%) at week 156 and 18 (58%) were in sustained clinical remission at weeks 52, 104 and 156 (Figure 6). An additional 2 (6%) patients were still on VDZ treatment at week 104 and 3 (10%) at week 156, respectively, but lacked information on the pMayo Clinic score within the pre-specified time-window.

**Figure 7.**
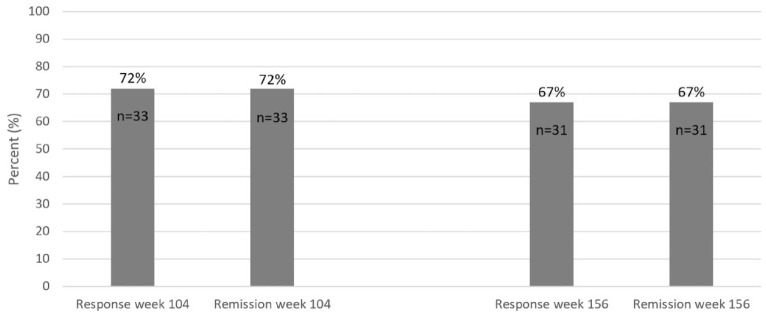
Clinical response and remission rates at weeks 104 and 156 in all UC patients (*n* = 46) in the SVEAH extension study. Missing data and discontinuation of VDZ were assumed to represent treatment failure. SVEAH, a Swedish observational study on vedolizumab assessing Effectiveness And Healthcare resource utilization in patients with inflammatory bowel disease; UC, ulcerative colitis.

Among all UC patients (*n* = 46) in the SVEAH extension study, 33 (72%) had a clinical response and 33 (72%) were in clinical remission at week 104 (Figure 7). Correspondingly, 31 (67%) patients had a clinical response and 31 (67%) were in clinical remission at week 156 (Figure 7). Data on the pMayo Clinic score were missing within the pre-specified time-window in three patients (7%) at week 104 and three patients (7%) at week 156. Among patients with concomitant systemic corticosteroids at baseline (*n* = 11), 6 (55%) were in corticosteroid-free remission at week 104 and 7 (64%) at week 156. In total, 45/46 (98%) UC patients in the SVEAH extension had a pMayo Clinic score at baseline: 37/40 (93%) patients also had a valid pMayo Clinic score at week 104 and 33/36 (92%) at week 156. Compared to baseline, the median (IQR) pMayo Clinic score decreased from 4 (3–5) to 0 (0–2) at week 104 (*p* < 0.001) and from 4 (3–5) to 0 (0–1) at week 156 (*p* < 0.001).

### Biochemical outcomes

All patients had information about P-CRP at baseline: 37 (93%) patients also had valid P-CRP values at week 104 and 34 (94%) patients at week 156. Another 3 (8%) patients still on VDZ lacked P-CRP data within the pre-specified time-window at week 104 and 2 patients (6%) at week 156. Compared to baseline, the median (IQR) P-CRP levels (mg/L) decreased from 5 (2–6) to 4 (1–5) at week 104 (*p* = 0.11) and from 5 (2–8) to 4 (2–4) at week 156 (*p* = 0.03). Correspondingly, the median (IQR) f-calprotectin concentrations (µg/g) decreased from 417 (174–787) to 51 (33–281) at week 104 (*p* = 0.003, *n* = 21) and from 387 (152–781) to 37 (21–275) at week 156 (*p* = 0.02, *n* = 17). The median (IQR) B-Hb concentration (g/L) increased from 136 (123–147) to 143 (129–151) at week 104 (*p* < 0.001, *n* = 38) and from 136 (123–147) at baseline to 145 (131–152) at week 156 (*p* = 0.005, *n* = 33).

### Endoscopic outcomes

Among UC patients (*n* = 46) in the SVEAH extension, 13 (28%) patients were in endoscopic remission, defined as a Mayo Clinic Endoscopic Subscore ⩽1, at week 104 and 10 (22%) patients at week 156. In total, 5 (11%) patients had signs of disease activity on endoscopy at week 104 and 3 (7%) patients at week 156. However, data on endoscopic activity were missing in 22 patients (48%) at week 104 and 23 (50%) patients at week 156. Therefore, we repeated the analyses and restricted the inclusion of patients to those with information on endoscopic outcomes at the follow-up visits. Among these patients, 72% (*n* = 13/18) achieved endoscopic remission at week 104 and 77% (10/13) at week 156.

### HRQoL outcomes

At baseline, 45 (98%) UC patients had information about the SHS. At week 104, all 40 patients still on VDZ had valid data on the SHS, and 33 (92%) patients at week 156. Compared to baseline, scores on the SHS improved significantly in all dimensions at weeks 104 and 156 (Table 4). Correspondingly, 39 (85%) patients had information about EQ-5D-5L at baseline. The median (IQR) EQ-5D-5L index value increased from 0.75 (0.69–0.80) at baseline to 0.86 (0.79–1.0) at week 104 (*p* < 0.001, *n* = 36) and from 0.79 (0.71–0.82) at baseline to 0.86 (0.79–1.0) at week 156 (*p* = 0.002, *n* = 30).

**Table 4.** HRQoL at baseline, at the start of VDZ treatment, at 104 and 156 weeks in patients with UC.

	Baseline	Week 104	*p*-value	Week 156	*p*-value
	Median (IQR)	Median (IQR)		Median (IQR)	
SHS					
Bowel symptoms	2 (2–3)	1 (0–1)	<0.001	1 (0–1)	<0.001
Activities of daily living	2 (1–3)	1 (0–1)	<0.001	1 (0–1)	<0.001
Worry	2 (1–3)	1 (0–2)	<0.001	1 (0–1)	<0.001
General well-being	2 (1–2)	1 (0–1)	<0.001	1 (0–1)	<0.001
EQ-5D-5L					
Mobility	1 (1–2)	1 (1–1)	0.083	1 (1–1)	0.705
Self-care	1 (1–1)	1 (1–1)	0.317	1 (1–1)	0.157
Usual activities	2 (1–3)	1 (1–1)	<001	1 (1–1)	0.018
Pain/discomfort	2 (2–3)	1 (1–2)	<0.001	1 (1–2)	0.001
Anxiety/depression	2 (1–3)	2 (1–2)	0.007	1 (1–2)	0.019
Visual analogue scale	70 (60–75)	85 (80–95)	<0.001	90 (80–95)	<0.001
EQ-5D-5L index value	0.76 (0.69–0.79)	0.86 (0.79–1.0)	<001	0.86 (0.79–1.0)	0.002

Comparisons between baseline and at week 104 (*n* = 36) and week 156 (*n* = 30) were performed using the Wilcoxon matched-pairs signed-rank test.

EQ-5D-5L, EuroQol 5-Dimensions 5-levels; HRQoL, health-related quality of life; IQR, interquartile range; SHS, Short Health Scale; UC, ulcerative colitis; VDZ, vedolizumab.

### Extraintestinal manifestations

Extraintestinal manifestations were observed in three UC patients (arthritis/arthralgia, *n* = 3) at baseline, five patients (arthritis/arthralgia *n* = 5) at week 104 and one patient (arthritis/arthralgia *n* = 1) at week 156.

### Predictors of remission

Univariate and multivariable logistic regression analyses were performed to identify predictors of clinical remission at week 156 (Table 5). Baseline clinical and demographical characteristics are presented in Supplementary Table 1. After adjustment for the potential confounders listed in Table 5, a high pMayo Clinic score (OR: 0.54; 95% CI: 0.38–0.76) was the only clinical variable at initiation of VDZ associated with clinical remission at week 156.

**Table 5.** Predictors of clinical remission at week 156 in patients with UC.

	Univariate analysis	Multivariable analysis
Female sex	1.20 (0.49–2.90)	0.68 (0.23–1.98)
Disease duration at baseline	1.02 (0.96–1.08)	1.03 (0.96–1.01)
pMayo Clinic score at baseline	**0.58 (0.43–0.79)**	**0.54 (0.38–0.76)**
Extent		
Proctitis (E1)	Ref	Ref
Left-sided colitis (E2)	Ref	Ref
Extensive colitis (E3)	1.80 (0.62–5.24)	3.13 (0.84–11.70)
Extraintestinal manifestations at baseline*	NA	NA
Previous surgery*	NA	NA
Concurrent medication at baseline		
Corticosteroids	1.14 (0.43–3.06)	0.57 (0.16–2.07)
Immunomodulators	0.81 (0.29–2.30)	1.03 (0.29–3.63)
Reasons for termination of last anti-TNF*	NA	NA

* Variables with <10 cases in each category were excluded.

Statistically significant results are highlighted in bold. anti-TNF, anti-tumour necrosis factor; pMayo Clinic score, partial Mayo Clinic score; UC, ulcerative colitis.

### Safety outcomes

No cases of malignancy or death occurred. Only one ADR was reported. A female patient discontinued VDZ because of coughing caused by an upper and lower respiratory infection.

### Sensitivity analyses

Patients participating in the SVEAH extension study were compared to patients in the initial SVEAH study who did not participate in the extension study to examine potential inclusion bias. Information about outcomes at week 52 (i.e. at the end of the SVEAH study), stratified for participation in the extension study or not, is provided in Supplementary Table 2. For patients with CD participating in the extension study, a higher remission rate at 12 weeks (*p* < 0.01) and a higher corticosteroid-free remission rate at 52 weeks were observed (*p* = 0.04). Similarly, a higher corticosteroid-free remission rate at 52 weeks was seen in UC patients in the SVEAH extension study compared to those who did not participate in the extension study (*p* = 0.02).

## Discussion

In this nationwide prospective observational multicentre extension of the SVEAH study, we report long-term outcomes in patients with CD or UC treated with VDZ. High clinical remission rates were observed at 3 years in CD (79%) and UC (71%) patients, who demonstrated an initial response to VDZ at 12 weeks. These findings were linked to improvements in HRQoL and high VDZ continuation rates at 3 years in patients with CD (84%) and UC (78%). Lower clinical disease activity, P-CRP concentrations and f-calprotectin levels were observed at the 3-year follow-up compared to the start of VDZ treatment. Few SAEs were reported.

Only two prospective real-world studies have examined response and remission status beyond 1 year of treatment.^28,29^ Amiot *et al.*^28^ reported outcomes up to 162 weeks in 294 French patients treated with VDZ. These authors found that remission rates decreased over time during treatment with VDZ. The clinical remission rate at the end of follow-up was 20% in patients with CD and 36% in patients with UC, and the corresponding corticosteroid-free clinical remission rates were identical.^28^ Similar rates were observed in a prospective study of 310 Dutch patients with IBD, where 20% of CD patients and 28% of UC patients were in clinical remission after 2 years.^29^ The corresponding rates for corticosteroid-free clinical remission were 19% and 28%, respectively. In the entire cohort of patients participating in the SVEAH extension study, 74% of patients with CD and 67% with UC were in remission at 3 years. The corresponding rates for corticosteroid-free remission were 36% and 64%, respectively. Comparisons of the results from the French and Dutch studies with our data must be interpreted cautiously because of different study designs. We included patients who re-consented at 52 weeks, that is, after 1 year of VDZ treatment. To generate representative results on corticosteroid-free remission rates, we only examined patients with concomitant corticosteroid treatment at the initiation of VDZ therapy (and few patients received corticosteroids at baseline). As a result, reported rates at 3 years were based on few events. In contrast to our study design, the French study examined remission rates up to 162 weeks among all patients who started VDZ therapy, whereas the Dutch study reported remission rates in patients with information on clinical measures at baseline and follow-up.^28,29^ Long-term outcomes of VDZ treatment have been reported from several additional real-world cohorts of IBD patients.^17,18^ However, most of these studies are limited by a retrospective design,^22,23,36^ analyses of patients at referral centres,^24^ lack of data beyond 1 year^19,20,37^ or only reports of VDZ persistence rates as long-term outcome measures.^21^

The observed remission rates in our study can be indirectly compared to some analyses of the GEMINI LTS cohort. In patients who continued VDZ therapy after 152 weeks, the clinical remission rate was 89% in those with CD (*n* = 62/70)^38^ and 96% in those with UC (*n* = 70/73).^39^ However, most patients in this programme had responded to induction therapy with VDZ because only responders at week 6 in the placebo-controlled randomized GEMINI trials were re-randomized to maintenance therapy. In addition, 37–52% of patients in the pivotal trials were naive to biologics. Conversely, in our cohort only a minority were naive to biologics (15% of the patients with CD and 9% with UC). Moreover, the dose regimen differed. In the GEMINI LTS, patients received VDZ 300 mg every 4 weeks, whereas the drug was administered according to the summary of product characteristics, that is, as an intravenous infusion of 300 mg every 8 weeks,^40^ in most of our patients. These variations probably explain some of the differences in remission rates between our cohort and the GEMINI LTS cohort.

When examining median f-calprotectin and P-CRP concentrations on the initiation of VDZ treatment in patients with CD and UC, we observed a decrease in f-calprotectin concentrations at both years 2 and 3 and decreased P-CRP levels at 3 years. These findings are partly supported by the studies on the association of f-calprotectin and P-CRP with long-term VDZ therapy, although conflicting data exist. In the GEMINI LTS trial, improvements in P-CRP concentrations were observed in patients with CD.^38^ Improved P-CRP levels have been observed in long-term follow-ups of some real-world cohorts of VDZ-treated patients with CD and UC.^26,41^ Still, most studies have failed to demonstrate a link between VDZ treatment and improvements in P-CRP concentrations^23^, or they have only observed an improvement in patients with CD.^25^ Long-term data on f-calprotectin in IBD patients treated with VDZ are limited. Attauabi *et al.* reported a significant decrease in f-calprotectin levels in 182 Danish patients after 52 and 104 weeks of VDZ treatment.^23^ The declines in f-calprotectin and P-CRP concentrations in our cohort were supported by improvements in endoscopic activity. However, these results should be interpreted carefully as many patients did not undergo endoscopy at years 2 and 3. Because we expect missing data on endoscopy during follow-up to be differential (i.e. degree depending on the exposure/outcome) and patients in remission with normalization of inflammatory markers to undergo endoscopy less often, this would underestimate the actual associations.

This study represents the first real-world effort to examine HRQoL measures beyond 1 year in IBD patients treated with VDZ. Because patients with IBD commonly experience decreased quality of life, HRQoL represents an important treatment goal in IBD. Therefore, HRQoL is included as an endpoint in clinical trial programmes. However, it is also important to examine HRQoL outcomes outside the setting of a placebo-controlled trial, where inclusion is restricted to a selected group of eligible patients. Based on the indices of HRQoL, we noted significantly increased HRQoL throughout the study period. We observed improvements in EQ-5D-5L and in all four SHS domains after 2 and 3 years. These novel results are reassuring for patients with CD and UC who start VDZ therapy in clinical practice. Our results are in line with the initial SVEAH study and confirm findings from the GEMINI 1 trial, in which VDZ treatment was associated with improvements in the EQ-5D visual analogue scale (VAS) and EQ-5D utility scores in UC patients compared to placebo.^42^ By contrast, Stallmach *et al.*^43^ only observed a statistically significant increase in the EQ-5D VAS from baseline to week 14, but not beyond in patients receiving VDZ.

In addition to the long-term data on HRQoL, other strengths of this study include the prospective multicentre design, standardization of data collection through an eCRF and assessment of validated outcome measures. Collectively, these measures increase the generalizability of our findings. The lack of a control group is a major limitation of the study, limiting the possibility of drawing firm conclusions about the efficacy of VDZ. Reported rates of various outcomes may underestimate the actual rates as we applied an intention-to-treat approach and treated missing data as treatment failure. Patients with missing data may also differ from those with complete data coverage. Patients with less severe disease are more likely to be followed less rigorously by the treating physician and less likely to have data reported during follow-up. Due to the nature of the study (observational design), endoscopy and clinical assessment were not compulsory at years 2 and 3. Although endoscopy is considered the gold standard to assess disease activity in IBD, many patients did not undergo endoscopic examinations within the pre-specified time-window (±8 weeks from the study visits at weeks 104 and 156) during follow-up or had missing data on endoscopy outcomes in SWIBREG. High disease activity at baseline was associated with a lower probability of remission at week 156 in both CD and UC, in line with the results at week 52 in our previous SVEAH study^27^, the GEMINI trials subgroup analyses^12,13^ and other real-world cohorts.^19,21,22,26,41,44^

In patients still treated with VDZ after 52 weeks in the original SVEAH cohort, 34% with CD and 42% with UC did not participate in the extension study. Statistically significant differences were observed when comparing these patients to those participating in the SVEAH extension. The corticosteroid-free remission rate at week 52 was higher in both CD and UC patients who participated in the extension study. Correspondingly, fewer patients with ileocolonic (L3) and penetrating (B3) CD were included in the extension study. In addition, HBI value at week 52 was lower among patients with CD who re-consented and took part in the extension study. Together, these differences indicate that patients with less severe disease participated in the SVEAH extension study. The extent to which these findings can be explained by differences in patients’ willingness to re-consent or investigators’ enthusiasm to invite patients to participate in the extension study is unclear. These results also show that re-consenting patients to long-term follow-up may introduce selection bias and may overestimate long-term outcomes.

## Conclusion

In conclusion, VDZ therapy demonstrated high long-term drug continuation rates and was associated with improvements in clinical disease activity, HRQoL measures and inflammatory markers during follow-up. Our findings support the use of VDZ in a real-world setting and confirm its role as a valid long-term treatment option in patients with moderate to severe UC and CD.

## Supplemental Material

sj-docx-1-tag-10.1177_17562848231174953 – Supplemental material for Long-term outcomes of vedolizumab in inflammatory bowel disease: the Swedish prospective multicentre SVEAH extension study Supplemental material, sj-docx-1-tag-10.1177_17562848231174953 for Long-term outcomes of vedolizumab in inflammatory bowel disease: the Swedish prospective multicentre SVEAH extension study by Isabella Visuri, Carl Eriksson, Sara Karlqvist, Byron Lykiardopoulos, Per Karlén, Olof Grip, Charlotte Söderman, Sven Almer, Erik Hertervig, Jan Marsal, Carolina Malmgren, Jenny Delin, Hans Strid, Mats Sjöberg, Daniel Bergemalm, Henrik Hjortswang and Jonas Halfvarson in Therapeutic Advances in Gastroenterology

sj-docx-2-tag-10.1177_17562848231174953 – Supplemental material for Long-term outcomes of vedolizumab in inflammatory bowel disease: the Swedish prospective multicentre SVEAH extension study Supplemental material, sj-docx-2-tag-10.1177_17562848231174953 for Long-term outcomes of vedolizumab in inflammatory bowel disease: the Swedish prospective multicentre SVEAH extension study by Isabella Visuri, Carl Eriksson, Sara Karlqvist, Byron Lykiardopoulos, Per Karlén, Olof Grip, Charlotte Söderman, Sven Almer, Erik Hertervig, Jan Marsal, Carolina Malmgren, Jenny Delin, Hans Strid, Mats Sjöberg, Daniel Bergemalm, Henrik Hjortswang and Jonas Halfvarson in Therapeutic Advances in Gastroenterology

sj-docx-3-tag-10.1177_17562848231174953 – Supplemental material for Long-term outcomes of vedolizumab in inflammatory bowel disease: the Swedish prospective multicentre SVEAH extension study Supplemental material, sj-docx-3-tag-10.1177_17562848231174953 for Long-term outcomes of vedolizumab in inflammatory bowel disease: the Swedish prospective multicentre SVEAH extension study by Isabella Visuri, Carl Eriksson, Sara Karlqvist, Byron Lykiardopoulos, Per Karlén, Olof Grip, Charlotte Söderman, Sven Almer, Erik Hertervig, Jan Marsal, Carolina Malmgren, Jenny Delin, Hans Strid, Mats Sjöberg, Daniel Bergemalm, Henrik Hjortswang and Jonas Halfvarson in Therapeutic Advances in Gastroenterology

## Declarations

**Ethics approval and consent to participate:** The study was approved by the Regional Ethics Review Board in Linköping (2014/375-31 with the amendment 2017/413-32). All eligible patients provided a separate written informed consent for this long-term follow-up before inclusion.

**Consent for publication:** Not applicable.

**Availability of data and materials:** The data underlying this article cannot be shared publicly for legal reasons, as they are obtained from a Swedish Quality Register. However, summary statistics can be provided on request.
