# Supplementary material for: Long-term outcomes of vedolizumab in inflammatory bowel disease: the Swedish prospective multicentre SVEAH extension study
Source: Ther Adv Gastroenterol. 2023 May 30;16:17562848231174953. doi: 10.1177/17562848231174953 (PMC10236258; doi:10.1177/17562848231174953)
Supplement: sj-docx-1-tag-10.1177_17562848231174953 – Supplemental material for Long-term outcomes of vedolizumab in inflammatory bowel disease: the Swedish prospective multicentre SVEAH extension study [file sj-docx-1-tag-10.1177_17562848231174953.docx]

# Appendix

The SWIBREG SVEAH Study Group consist of the following collaborators: Marie Andersson^1^, Jonas Bengtsson^2^, Lars-Åke Bark^3^, Jan Björk^4-5^, Michael Eberhardson^6^, Per Hellström^7^, Joakim Holmin^8^, Ulrika L. Fagerberg^9-11^, Jenny Gunnarsson^12^ Susanna Jäghult^13^, Pontus Karling^14^, Jonas F Ludvigsson^15-18^, Pär Myrelid^19-20^, Caroline Nordenvall^21-22^, Carin Ocklind^23^, Ola Olén^24-25^, Malin Olsson^20^, Mikael Olsson^26^, Martin Rejler^27-28^, Daniel Sjöberg^29^, Henrik Stjernman^30^, Otto Überbacher^31^, David Öberg^32^

Affiliations for The SWIBREG SVEAH Study Group:

(1) Department of Internal Medicine, Södra Älvsborgs Hospital, Borås, Sweden.

(2) Department of Surgery, Sahlgrenska University Hospital/Östra, Gothenburg, Sweden.

(3) Department of Internal Medicine, Södersjukhuset, Stockholm, Sweden

(4) Unit of Internal Medicine, Institute Medicine Solna, Karolinska Institutet, Stockholm, Sweden.

(5) Patient Area Gastroenterology, Dermatovenerology and Rheumatology, Inflammation and Infection Theme Karolinska University Hospital, Stockholm, Sweden.

(6) Department of Medicine Solna, Karolinska Institutet, Stockholm, Sweden.

(7) Department of Medical Sciences, Gastroenterology and Hepatology unit, Uppsala University, Uppsala, Sweden.

(8) Department of Internal Medicine, Halmstad Hospital, Halmstad, Sweden

(9) Center for Clinical Research, Västmanland Hospital, Västerås, Sweden and Uppsala University, Uppsala, Sweden.

(10) Department of Pediatrics, Västmanland Hospital, Västerås, Sweden.

(11) Department of Women´s and Children´s Health, Karolinska Institutet, Stockholm, Sweden.

(12) Department of Internal Medicine, Kungälv Hospital, Kungälv, Sweden

(13) Stockholm Gastro Center, Karolinska Institutet, Stockholm, Sweden.

(14) Department of Public Health and Clinical Medicine, Umeå University, Umeå, Sweden.

(15) Department of Medical Epidemiology and Biostatistics, Karolinska Institutet, Stockholm, Sweden

(16) Department of Pediatrics, Örebro University Hospital, Örebro University, Örebro, Sweden

(17) Division of Epidemiology and Public Health, School of Medicine, University of Nottingham, Nottingham, UK.

(18) Department of Medicine, Celiac Disease Center, Columbia University College of Physicians and Surgeons, New York, NY, USA

(19) Department of Clinical and Experimental Medicine, Linköping University, Linköping, Sweden

(20) Department of Surgery, County Council of Östergötland, Linköping, Sweden.

(21) Department of Molecular Medicine and Surgery, Karolinska Institutet, Stockholm, Sweden.

(22) Department of Pelvic Cancer, Colorectal surgery unit, Karolinska University Hospital, Stockholm, Sweden.

(23) Department of Internal Medicine, Västmanland Hospital, Västerås, Sweden

(24) Clinical Epidemiology Division, Department of Medicine Solna, Karolinska Institute, Stockholm, Sweden

(25) Sachs' Children and Youth Hospital, Department of Clinical Science and Education, Stockholm South General Hospital, Karolinska Institute, Stockholm, Sweden

(26) Department of Internal Medicine, Norra Älvsborgs Hospital, Trollhättan, Sweden

(27) Department of Medicine, Höglandssjukhuset Eksjö, Region Jönköping County Council, Jönköping, Sweden.

(28) Jönköping Academy for Improvement of Health and Welfare, Jönköping University, Jönköping, Sweden

(29) Center of Clinical Research in Dalarna, Falun, Sweden.

(30) Department of Medicine, County Hospital Ryhov, Jönköping, Sweden

(31) Department of Internal Medicine, Hallands Hospital, Varberg, Sweden.

(32) Department of Internal Medicine, Sunderby Hospital, Sunderbyn, Sweden.
