# Supplementary material for: Long-term outcomes of vedolizumab in inflammatory bowel disease: the Swedish prospective multicentre SVEAH extension study
Source: Ther Adv Gastroenterol. 2023 May 30;16:17562848231174953. doi: 10.1177/17562848231174953 (PMC10236258; doi:10.1177/17562848231174953)
Supplement: sj-docx-2-tag-10.1177_17562848231174953 – Supplemental material for Long-term outcomes of vedolizumab in inflammatory bowel disease: the Swedish prospective multicentre SVEAH extension study [file sj-docx-2-tag-10.1177_17562848231174953.docx]

**Supplementary Table 1.** Demographics and clinical characteristics at initiation of vedolizumab treatment in patients with Crohn's disease and ulcerative colitis included in the SVEAH extension study, and patients who discontinued vedolizumab treatment before week 52 in the SVEAH study

|  | **Crohn's disease (n=134)** | **Ulcerative colitis (n=84)** |
| --- | --- | --- |
| **Female sex, n (%)** | 67 (50) | 41 (49) |
| **Median age, years (IQR)** | 43 (29-53) | 42 (27-53) |
| **Disease duration, years (IQR)** | 10 (4-21) | 6 (3-11) |
| **Smoker, n (%)** | 18 (13) | 4 (5) |
| **Median HBI (IQR)** | 6 (3-9) |  |
| **Median pMayo score (IQR)** |  | 5 (3-6) |
| **Disease location, n (%)** |  |  |
| Ileal, L1 | 22 (16) |  |
| Colonic, L2 | 47 (35) |  |
| Ileocolonic, L3 | 65 (49) |  |
| Isolated upper disease, L4 | 0 (0) |  |
| **Disease behaviour, n (%)** |  |  |
| Inflammatory, B1 | 78 (58) |  |
| Stricturing, B2 | 44 (33) |  |
| Penetrating, B3 | 12 (9) |  |
| Perianal, p | 27 (20) |  |
| **Disease extent, n (%)** |  |  |
| Proctitis, E1 |  | 1 (1) |
| Left-sided colitis, E2 |  | 21 (25) |
| Extensive colitis, E3 |  | 62 (74) |
| **Previous biologics, n (%)** |  |  |
| 0 | 16 (12) | 8 (10) |
| 1 | 53 (40) | 47 (56) |
| ≥2 | 65 (49) | 29 (35) |
| **Reason for termination of last biological treatment, n (%)** |  |  |
| Primary non-response | 25 (21) | 28 (37) |
| Loss of response | 50 (42) | 33 (43) |
| Intolerance | 36 (31) | 13 (17) |
| Other reasons | 7 (6) | 2 (3) |
| **Previous IBD surgery, n (%)** | 57 (43) | 7 (8)^a^ |
| **Extraintestinal manifestations, n (%)** | 31 (23) | 8 (10) |
| **Concomitant medication, n (%)** |  |  |
| 5-aminosalicylic acid | 8 (6) | 32 (38) |
| Corticosteroids | 26 (19) | 23 (27) |
| Immunomodulators | 16 (12) | 21 (25) |

*^a^Colectomy with ileorectal anastomosis. IQR, Interquartile range; HBI, Harvey Bradshaw index; pMayo score, Partial Mayo Clinic score; IBD, Inflammatory bowel disease*
