# Supplementary material for: Long-term outcomes of vedolizumab in inflammatory bowel disease: the Swedish prospective multicentre SVEAH extension study
Source: Ther Adv Gastroenterol. 2023 May 30;16:17562848231174953. doi: 10.1177/17562848231174953 (PMC10236258; doi:10.1177/17562848231174953)
Supplement: sj-docx-3-tag-10.1177_17562848231174953 – Supplemental material for Long-term outcomes of vedolizumab in inflammatory bowel disease: the Swedish prospective multicentre SVEAH extension study [file sj-docx-3-tag-10.1177_17562848231174953.docx]

Supplementary Table 2. Demographic, clinical characteristics and outcomes at baseline, week 12 and week 52 in patients included and patients eligible for the SVEAH extension study.

|  | **Extension (CD) (n=68)** | **Eligible but not in extension (CD) (n=35)** | **P-value*** | **Extension (UC) (n=46)** | **Eligible but not in extension (UC) (n=33)** | **P-value*** |
| --- | --- | --- | --- | --- | --- | --- |
| **Female sex, n (%)** | 27 (40) | 17 (49) | .39 | 23 (50) | 10 (30) | .08 |
| **Median age at baseline (IQR)** | 43 (31-53) | 47 (33-64) | .42 | 42 (26-53) | 38 (25-63) | .89 |
| **Disease duration (IQR)** | 9 (3-23) | 9 (5-22) | .53 | 6 (3-11) | 5 (2-14) | .95 |
| **Smoker, n (%)** | 8 (12) | 5 (14) | .72 | 2 (4) | 1 (3) | .76 |
| **Disease location,  n (%)** |  |  |  |  |  |  |
| Ileal, L1 | 16 (23) | 6 (17) | .45 |  |  |  |
| Colonic, L2 | 24 (35) | 5 (14) | **.03** |  |  |  |
| Ileocolonic, L3 | 28 (41) | 23 (66) | **.02** |  |  |  |
| Isolated upper disease, L4 | 0 (0) | 1 (3) |  |  |  |  |
| **Disease behaviour, n (%)** |  |  |  |  |  |  |
| Inflammatory, B1 | 42 (62) | 10 (29) | **<.01** |  |  |  |
| Stricturing, B2 | 21 (31) | 16 (46) | .14 |  |  |  |
| Penetrating, B3 | 5 (7) | 9 (26) | **.01** |  |  |  |
| Perianal, p | 13 (19) | 9 (26) | .44 |  |  |  |
| **Disease extent, n (%)** |  |  |  |  |  |  |
| Proctitis, E1 |  |  |  | 1 (2) | 1 (3) | .81 |
| Left-sided colitis, E2 |  |  |  | 9 (20) | 7 (21) | .86 |
| Extensive colitis, E3 |  |  |  | 36 (78) | 25 (76) | .79 |
| **Previous biologics, n (%)** |  |  |  |  |  |  |
| 0 | 10 (15) | 3 (9) | .38 | 4 (9) | 8 (24) | .06 |
| 1 | 24 (35) | 13 (37) | .85 | 28 (61) | 11 (33) | **.02** |
| >2 | 34 (50) | 19 (54) | .68 | 14 (30) | 14 (42) | .27 |
| **Reason for termination of last biological treatment, n (%)** |  |  |  |  |  |  |
| Primary non-response | 13 (22) | 5 (16) | .44 | 15 (36) | 10 (40) | .83 |
| Loss of response | 27 (47) | 10 (31) | .16 | 19 (45) | 10 (40) | .32 |
| Intolerance | 15 (26) | 11 (34) | .39 | 8 (19) | 3 (12) | .29 |
| Other reasons | 3 (5) | 6 (19) | **.04** | 0 (0) | 2 (8) | .09 |
| **Previous IBD surgery, n (%)** | 27 (40) | 13 (37) | .80 | 2 (4) † | 2 (6) † | .73 |
| **Concomitant medication, n (%)** |  |  |  |  |  |  |
| 5-ASA | 4 (6) | 3 (9) | .61 | 20 (43) | 11 (33) | .36 |
| Corticosteroids | 14 (21) | 5 (14) | .44 | 11 (24) | 3 (9) | .09 |
| Immunomodulators | 10 (15) | 4 (11) | .65 | 11 (24) | 7 (21) | .78 |
|  |  |  |  |  |  |  |
| **Response week 12,  n (%)** | 23 (34) | 11 (31) | .81 | 25 (54) | 18 (55) | .99 |
| **Remission week 12,  n (%)** | 46 (68) | 13 (37) | **<.01** | 22 (48) | 9 (27) | .07 |
| **Response week 52,  n (%)** | 25 (37) | 13 (37) | .97 | 32 (70) | 23 (70) | .99 |
| **Remission week 52,  n (%)** | 50 (74) | 19 (54) | .05 | 34 (74) | 17 (52) | **.04** |
| **Corticosteroid-free remission week 52, n (%)** | 49 (72) | 18(51) | **.04** | 34 (74) | 16 (48) | **.02** |
| **HBI week 52,  median (IQR)** | 2 (1-4) | 4 (2-8) | **.03** |  |  |  |
| **Partial Mayo Clinic score week 52,  median (IQR)** |  |  |  | 1 (0-2) | 1 (0-2) | .23 |
| **F-calprotectin week 52, median (IQR)** | 90 (65-300) | 168 (58-598) | .47 | 68 (35-304) | 168 (52-421) | .31 |
| **P-CRP week 52, median (IQR)** | 3 (2-5) | 4 (2-11) | .21 | 4 (1-5) | 4 (2-8) | .23 |
| **B-Hb week 52, median (IQR)** | 140 (131-147) | 133 (127-144) | .12 | 139 (132-150) | 144 (134-156) | .35 |
| **Extraintestinal manifestations,  week 52** | 15 (22) | 10 (29) | .47 | 8 (17) | 5 (15) | .79 |
| **SHS at week 52,  median (IQR)** | 4 (2-7) | 6 (3-8) | **.05** | 4 (2-5) | 4 (2-6) | .98 |
| **EQ5D-5L index value at week 52,  median (IQR)** | 0.86 (0.75-1.00) | 0.79 (0.73-0.86) | .15 | 0.86 (0.79-1.00) | 0.86 (0.78-1.00) | .91 |
| Abbreviations: IQR, interquartile range; anti-TNF, anti-tumour necrosis factor; HBI, Harvey-Bradshaw index; f-calprotectin, faecal calprotectin; P-CRP, Plasma C-reactive protein; B-Hb, Blood Haemoglobin; SHS, Short health scale; EQ5D-5L, EuroQoL 5 dimensions 5 levels  Bold values denote statistical significance at the p <0.05 level  *Mann-Whitney U or Chi-2 test  †Colectomy with ileorectal anastomosis | | | | | | |
